# Treatment Strategies in Advanced-Stage Hodgkin Lymphoma

**DOI:** 10.3390/cancers16112059

**Published:** 2024-05-29

**Authors:** Eldad J. Dann, René-Olivier Casasnovas

**Affiliations:** 1 Department of Hematology and Bone Marrow Transplantation, Rambam Health Care Campus, Haifa 3109601, Israel; 2 Blood Bank and Apheresis Unit, Rambam Health Care Campus, Haifa 3109601, Israel; 3 The Ruth and Bruce Rappaport Faculty of Medicine, Technion, Israel Institute of Technology, Haifa 3109601, Israel; 4 Department of Hematology, CHU Dijon Bourgogne, 21000 Dijon, France; olivier.casasnovas@chu-dijon.fr; 5 INSERM 1231 Team Epi2THM ((Epi)genetics, Epidemiology and Targeted Therapy in Hematological Malignancies), 21000 Dijon, France

**Keywords:** advanced Hodgkin lymphoma, interim PET/CT, international prognostic scoring, anti-PD1, brentuximab vedotin

## Abstract

**Simple Summary:**

The treatment of advanced-stage Hodgkin lymphoma (HL) is a rapidly evolving field with the introduction of new antibody-mediated drugs to first-line therapies and improving progression-free survival (PFS) to 80–94%. The novel modalities include the anti-CD30 antibody drug conjugate brentuximab vedotin and the anti-PD1 antibody (immune checkpoint inhibitor) that blocks a receptor, helping HL cells to turn off the immune cells, fighting malignant cells. When aggressive therapy is used, a negative result of the interim PET/CT scan can be predictive of a favorable outcome, allowing for a subsequent de-escalation of therapy. A more aggressive therapy is associated with higher short-term and long-term toxicity. Thus, the treatment choice for young patients (median age 30–34 years) should also take into consideration the long-term toxicity, since their expected life longevity exceeds half a century. The current review analyzes the recently published studies that may become practice changing and lead to significantly improved PFS.

**Abstract:**

The last 3 decades have witnessed a major evolution in the treatment of advanced-stage Hodgkin lymphoma (HL). The most prominent of these developments include the introduction of the international prognostic scoring (IPS) system; therapeutic decision-making based on both IPS and interim PET/CT data; the finding that a negative interim PET/CT result could be safely used for treatment de-escalation; the introduction of intensive combination chemotherapy like escalated BEACOPP (bleomycin, etoposide, adriamycin, cyclophosphamide, oncovin (vincristine), procarbazine, and prednisone); and further modification of this protocol with the incorporation of a conjugated anti-CD30 antibody brentuximab vedotin (BV) into first-line regimens, like BV-AVD (BV+ adriamycin, vinblastine and dacarbazine) and BrECADD (brentuximab vedotin, etoposide, cyclophosphamide, doxorubicin, dacarbazine, and dexamethasone). The accruing data about the toxicity of the escalated BEACOPP protocol have led to decreasing the number of therapeutic cycles, substitution of toxic agents like procarbazine with dacarbazine (e.g., BEACOPDac), and reduction/omission of radiation therapy. Lately, a significant advancement has been made by the integration of checkpoint inhibitors in the first-line treatment, with preliminary results demonstrating the superiority of anti-PD1 combined with chemotherapy (nivolumab-AVD) compared to the BV-AVD regimen. This review aims to analyze recently published studies whose findings could change the treatment practice in advanced-stage HL.

## 1. Introduction

The pioneering, multi-agent chemotherapy approach to the treatment of solid tumors was first employed in patients with advanced-stage Hodgkin lymphoma (HL). In the 1960s, the team from the National Cancer Institute (NCI) in the United States introduced the MOPP regimen, including mechlorethamine hydrochloride (mustard), oncovin (vincristine), procarbazine, and prednisone, that could offer a cure for about 50% of patients with advanced disease [1]. This brought a pivotal shift in the treatment concept from a single-agent to a multi-agent therapeutic strategy. Yet, MOPP was associated with significant toxicity, including neurotoxicity up to paraparesis related to the non-restricted dosage of vincristine (up to 4 mg), secondary leukemia, late solid tumors, and sterility in males and some females. To address these issues, the research group from Milano led by G. Bonadonna developed the ABVD (adriamycin, bleomycin, vinblastine, and dacarbazine) regimen, given on days 1 and 15 of each cycle [2]. The latter treatment did not affect fertility and was considered safe in terms of secondary leukemia. In a randomized study by G. Canellos et al., including 361 patients who were randomized to 3 arms to receive 6–8 cycles of MOPP or ABVD or an alternating regimen of MOPP-ABVD, complete response (CR) rates were 67%, 82%, and 83%, respectively [3]. The 5-year failure-free survival (FFS) was 50%, 61%, and 65%, respectively. Although no overall survival (OS) superiority was demonstrated due to successful salvage of patients, the ABVD regimen became the backbone of HL therapy and remained as such for 3 decades, with current FFS values getting close to 80%. This achievement could be explained by the established strict timetable of treatment administration, irrespective of neutropenia that could be reduced using the granulocyte colony-stimulating factor (G-CSF). Nowadays, new treatment paradigms, aiming at improvement of FFS and reduction in late toxicity, are rapidly evolving.

## 2. Intensification of Therapy for Patients with Newly-Diagnosed Advanced-Stage HL

In 1998, the German Hodgkin Study Group (GHSG), led by V. Diehl, introduced a more aggressive protocol than ABVD [4]. In that randomized controlled trial (HD9), comparing three chemotherapy regimens, the protocol termed “escalated BEACOPP” (EB) was found to be associated with a significantly higher FFS of 87% relative to 76% for standard BEACOPP (SB) and 69% for COPP-ABVD (cyclophosphamide, vincristine, procarbazine, and prednisone), with a 5-year OS of 91%, 88%, and 83%, respectively. Ninety-eight percent of the patients suffered grade 3–4 neutropenia and seventy percent experienced grade 3–4 thrombocytopenia. It is noteworthy that radiation therapy was applied to 70% of patients from the BEACOPP arms and to 64% of patients from the COPP-ABVD arm [5]. The EB protocol remained the mainstay of advanced-stage HL therapy in Germany and several other countries for patients up to the age of 60 years for two decades. A head-to-head comparison between EB and ABVD was conducted by the Gruppo Italiano di Terapie Innovative nei Linfomi (GITIL) and the Intergruppo Italiano Linfomi, demonstrating a significantly better 7-year FFP of 85% versus 73% (*p* = 0.004) for EB compared to ABVD. However, neither the 7-year freedom from a second progression (88% versus 82%; *p* = 0.12) nor the 7-year OS (89% versus 84%; *p* = 0.39) were found to significantly differ between the two regimens [6]. Based on these findings, 6–8 cycles of ABVD remained the standard treatment in the majority of countries, as the FFS benefit observed with EB relative to ABVD did not translate into a benefit in OS. In 2020, a pooled analysis of mature data on 1227 patients from four European randomized controlled trials (RCTs), comparing aggressive therapy (4 EB + 2–4 SB versus 6–8 ABVD), showed the superiority of the 7-year progression-free survival (PFS) in the BEACOPP arm (81.1%) relative to the ABVD arm (71.1%; *p* < 0.001) [7]. Notably, the OS difference became significant at a follow-up of >18 months (93.2% versus 88.3%, respectively).

## 3. Treatment Modifications Based on Interim PET/CT

In the early 2000s, another major advancement was made with the introduction of an interim assessment of disease response to therapy based on functional imaging using PET/CT, which is performed following 2 cycles of treatment, as a predictive factor for patient outcome [8]. It is still a matter of debate if all patients with advanced disease require aggressive therapy or if it needs to be considered only for a subset of patients with high international prognostic scoring (IPS) [9]. In a multivariate analysis of data from the four aforementioned RCTs, performed at >18 months post-randomization, both the type of the therapeutic regimen and high IPS (≥3) were found to be significant for OS [7]. Phase 2 studies by the Israeli Hodgkin study group introduced a different approach to the initial management of patients with lower-risk (IPS 0–2) and higher-risk (IPS ≥ 3) advanced-stage HL as well as implemented treatment modifications based on the results of interim PET (PET-2) conducted after 2 therapeutic cycles. In the latter studies, patients with IPS 0–2 first received SB or ABVD, and in individuals with positive PET-2, the therapy was escalated. Patients with IPS ≥3 initiated therapy with EB, and in those of them who had negative PET-2, the treatment was de-escalated to either SB or ABVD [10,11]. At the same time, both in the Risk-Adapted Therapy in Hodgkin Lymphoma (RATHL) trial [12] and the GITIL/FIL HD 0607 trial [13], treatment was started with 2 cycles of ABVD for all patients, irrespective of their IPS, and was then escalated to 4 cycles of EB ± 4 cycles of SB for patients with positive PET-2, defined as the Deauville score 4–5 [14,15] by central expert reading (Table 1). The 3-year PFS for patients with negative and positive PET-2 was 85.7% and 67.5% in the RATHL trial and 87% and 60% in the GITIL trial [12,13]. A comparable 4-year PFS of 65% was observed in PET-2-positive patients in the Israeli H2 study [11]. In the S0816 trial by the US Southwest Oncology Group (SWOG), all patients started therapy with ABVD, and in those with positive PET-2, treatment was changed to 6 cycles of EB. The 5-year PFS amounted to 76% and 66% for patients with negative and positive PET-2 results, respectively [16]. Zinzani et al. in the HD0801 trial also initiated therapy with 2 cycles of ABVD, and patients with positive PET-2 (about 20%) received salvage therapy followed by autologous stem cell transplantation (SCT) [17]. In that group, incorporating 103 patients, 85 actually underwent SCT. In the intention-to-treat analysis, the 2-year PFS was found to be 76% in PET-2-positive patients compared to 81% in PET-2-negative patients. In our opinion, since the majority of patients could be salvaged with the EB-type regimen, SCT should be reserved only for patients with positive PET-4 or for those experiencing disease progression while on EB therapy. The achieved PFS in PET-2-positive patients was comparable in the GITIL, RATHL, and S0816 studies, suggesting the lack of advantage in using more than 4 additional EB cycles in this patient population.

The RATHL and GITIL/FIL HD 0607 studies provided further important findings [12,13]. In the RATHL study, patients with negative PET-2 were randomized to continue ABVD or AVD, showing a PFS difference of 1.6% (NS), which gave the rationale for omitting bleomycin for such patients in order to prevent pulmonary toxicity. Moreover, no radiation therapy was given to patients with negative PET-2, which was associated with a favorable 3-year PFS of around 85%. Based on these findings, the 2022 guidelines by the British Society for Haematology recommended the exclusion of bleomycin from the remaining 4 ABVD cycles, i.e., using AVD instead [18].

The issue of applying radiation therapy as consolidation for patients with a bulky disease and negative PET-2 was addressed in the HD0801 and GITIL studies. In the GITIL study, the randomization of patients with both interim and end-of-therapy negative PET demonstrated that radiation therapy to bulky masses ≥5 cm did not add a significant benefit to PFS and, thus, could be omitted. Similar conclusions were reached in the HD0801 study [19]. Likewise, in the HD15 trial [20], where upfront EB was administered to all patients, additional radiation therapy was limited to patients who were PET positive at the end of therapy and had a persistent residual mass >2.5 cm (11%). Based on these data, radiation therapy is no longer an integral part of the treatment for advanced-stage HL and is reserved for the minority of patients who are suspected of achieving partial response.

The data obtained from the trials where treatment was initiated with ABVD demonstrated that patients with positive PET-2 benefitted from therapy escalation; however, the PFS was yet inferior to that observed in patients who achieved early metabolic response (negative PET-2). A more intensive approach to the management of all patients with advanced-stage HL or those with stage IIB bulky disease was introduced by the GHSG [5]. In the HD18 study, the initial therapy included 2 cycles of EB followed by PET-2 [21]. Patients who had a negative PET-2 (Deauville score < 3) were randomized to additional 2 cycles versus 4 cycles of EB (a total of 4 versus 6–8 cycles). In that study, the 5-year estimated PFS was 91.4 (95% CI 89.5–93.4) and 89.3 (95% CI 85.8–90.8) for patients with negative and positive PET-2, respectively, evaluated based on the criteria of PET-2 positivity, including the Deauville score 3. However, since studies by other groups interpreted the Deauville score 3 as a negative PET result, a sub-analysis was conducted in the HD18 study for patients with Deauville score 3 who received 6 cycles of EB. Since the obtained results were comparable to those found in patients with the Deauville score 1–2, the score 1–3 was adopted as a relevant interpretation criterion for PET-2 negativity in subsequent GHSG studies [21]. The HD18 study suggested that for patients with negative PET-2, an overall of 4 EB cycles could become the new standard of care. Notably, the 3-year PFS of patients with negative and positive PET-2, initially treated with EB, was about 10% and 20% higher than for those who started therapy with ABVD (Table 1).

Despite such promising results, treatment-related toxicity remains one of the major concerns, which makes its management the primary focus of numerous current studies. The phase 3 LYSA AHL2011 trial, including patients from 90 centers in Belgium and France, evaluated the effect of de-escalation of therapy after 2 cycles of EB to 4 cycles of ABVD [22] in PET-2-negative patients in the experimental arm, while 4 cycles of EB were maintained for PET-2-positive patients. In the standard treatment arm, where therapy was not PET-guided, all the patients received 6 EB cycles. The study also included a second interim PET performed following 4 cycles of chemotherapy (PET-4). Similar outcomes in terms of PFS and OS were demonstrated in both arms. The group of PET-2- and PET-4-negative patients reached a 5-year PFS of 92.5 (95% CI 90.1–94.3), whereas the 5-year PFS for PET-2-positive and PET-4-negative patients was 75.4% (95% CI 62.5–84.4). The 5-year PFS was significantly lower in patients with positive PET-4, amounting to only 46.5% (95% CI 31.2–60.4). In a multivariate analysis, PET-2 positivity and PET-4 positivity were identified as significant risk factors for disease progression, with a hazard ratio (HR) of 3.59 (95% CI 2.01–6.04) and 13.14 (95% CI 7.31–19.51), respectively. Likewise, the IPS > 3 was found to be a significant risk factor for HL progression (HR 1.92; 95% CI 1.24–2.94) [23]. That seminal study demonstrated several important results. First, IPS remains a valid parameter when the treatment is started with more aggressive therapy, as patients with a high score are doing less well. Furthermore, the positivity of PET-2 and especially of PET-4 is a strong predictor for an inferior PFS. Finally, therapy can be safely de-escalated in patients with negative PET-2. This finding is of high importance, given that while the aforementioned studies use upfront EB based on the “Kairos” principle and show improved PFS for the whole group, this comes at the expense of increased rates of grade 3–4 neutropenia and reduced fertility. The PET-guided strategy applied in the AHL2011 study significantly reduces the risk of infertility, including premature ovarian insufficiency, by decreasing the number of EB cycles from 6 to 2 [24]. 

**Table 1:** Recent studies of treatment of advanced-stage Hodgkin lymphoma.

Study	Age, Years	Disease	PET-Adapted	Protocol	No. of	PFS,	OS,
		Stage			Patients	%	%
RATHL [12]	≥18	IIB–IV	Yes		1203	All, 3-y: 82.6 (80.6–84.7)	All, 3-y: 95.8 (94.4–96.8)
			PET-2 pos (DS 4, 5): 16.3%	ABVD x2	172	3-y: 67.5	3-y: 87.8
				+ EB x4			
			PET-2 neg: 83.7%	ABVD x2 +	465	3-y: 84.4	3-y: 97.6
				AVD x4			
				v	v	v	v
				ABVD x2 + ABVD x4	470	85.7	97.2
GITIL HD0607 [13]	14–60	IIB–IVB	Yes		780	All, 3-y: 82	All, 3-y: 97
			PET-2 pos (DS 4, 5): 19%	ABVD x2 + EB x4 + SB x4 ± rituximab	150	3-y: 60	3-y: 89
			PET-2 neg: 81%	ABVD x2 + ABVD x4 ± RT to mass ≥5	630	3-y: 87	3-y: 99
SWOG S0816 [16]	18–60	III–IV	Yes		331	All, 5-y: 74	All, 5-y: 94
			PET-2 pos (DS 4, 5): 18%	ABVD x2 + EB x6	61	5-y: 66	5-y: 86
			PET-2 neg: 82%	ABVD x2 + ABVD x4	270	5-y: 76	5-y: 96
ECHELON-1 [25]	15–60	III–IV	No	BV-AVD x6	664	6-y: 82.3% (79.1–85.0)	6-y: 93.9% (91.6–95.5)
				v	v	v	v
				ABVD x6	670	74.5% (70.8–77.7)	89.4% (86.6- 91.7)
			PET-2 pos (DS 4, 5): 8%	BV-AVD x6 v ABVD x6	47 v 58	6-y: 61 (45–73) v 46 (33–58)	6-y: 95 v 77
			PET-2 neg: 92%		588 v 577	6-y: 85 (81.8–87.7) v 78.1 (74.3–81.3)	6-y: 94.9 v 90.6
SWOG S1826 [26]	≥12	III–IV	No		976		
				Nivo-AVD x6	489	1-y: 94 (91–96)	1-y: 99 (98–100)
				v	v	v	v
				BV-AVD x6	487	1-y: 86 (82–90)	98 (94–99)
LYSA AHL2011 [23]	16–60	IIB–IV	Yes		823	5-y: 87.5–86.7	5-y: 97.7
			Standard arm (non-PET-driven therapy)	EB x6	413		
			PET-2 pos		49	5-y: 73.5 (58.7–83.6)	5-y: 93.7% (81.7–97.9)
			PET-2 neg		349	5-y: 89.9% (86.2–92.7)	97.6% (95.2–98.8)
			PET-Driven Arm		397		
			PET-2 pos (DS 4, 5): 12.6%	EB x2 + EB x2			
			PET-4 neg	+ EB x2	35	5-y:75% (62.6 to 84.4)	5-y: 93.5%
			PET-4 pos	+ salvage	16	5-y: 46.5% (31.2–60.4)	93%
			PET-2 neg: 84%	EB x2+ ABVD x4	346	5-y: 90.5% (86.9–93.2)	5-y: 98%
HD18 [21]	18–60		Yes		1945		
			PET-2 pos (DS 3–5)	EB x8 or R-EB x8	434	5-y: 89.7% (85.4–94.0) or 88.1% (83.5–92.7)	5-y: 96.4% (93.8–99.0) or 93.9% (90.6–97.3)
			PET-2 neg (DS 1–2): Standard arm	EB x8 or EB x6	504	5-y: 90.8% (87.9–93.7)	3-y: 97.5% (95.2–99.7)
			Experimental arm	EB x4	501	5-y: 92.2% (89.4–95.0)	3-y: 98.8% (97.2–100.0)
HD21 [27,28]	18–60	IIB–IV	Yes		1482		
			PET-2 pos (DS 4, 5): 41%	EB x6 v	740	3-y: 90.6% (87.1–94.1) v	3-y: 98.5
				BrECADD x6		3-y: 93.5% (90.6–96.5)	
			PET-2 neg (DS 1–3): 51%	EB x4 v	742	3-y: 93.6% (91.3–95.9) v	
				BrECADD x4		3-y: 97.1% (95.5–98.7)	

DS: Deauville score; SB: standard BEACOPP; EB: escalated BEACOPP.

## 4. Incorporation of Antibodies in the First-Line Treatment Regimens for Advanced-Stage HL

Further progress in the management of advanced-stage HL was associated with the inclusion of the anti-CD30 antibody drug conjugate brentuximab vedotin (BV) and the anti-PD1 antibody (immune checkpoint inhibitors, like nivolumab or pembrolizumab) in therapeutic protocols. These novel modalities changed the standard of care in this disease. The results of the seminal ECHELON-1 phase 3 trial, conducted by JM Connors et al., that compared BV-AVD to ABVD, demonstrated a significantly better PFS in patients treated with the former regimen [29]. However, the documented 5% superiority in the 2-year modified PFS (82.1% versus 77.2%) came at the expense of significantly higher rates of grade ≥2 peripheral neuropathy (31% versus 11%) and neutropenia (58% versus 45%). At a longer follow-up, improved 6-year PFS (82.3% versus 74.5%) and OS (93.9% versus 89.4%) were recorded [25]. In the BV-AVD group, 32/39 deaths were either HL or therapy related. In ABVD-treated patients, the corresponding values equated to 45/64 deaths; among the 19 additional deaths, 11 were caused by a second malignancy. Importantly, in the BV-AVD arm, a significantly superior PFS was documented in patients with IPS ≥ 4 and those with stage IV disease. This finding led to the registration of BV by the European Medicines Agency (EMA) as the first-line therapy for patients with stage IV disease. The results obtained demonstrated that, for patients with high-risk HL (high IPS or stage IV), ABVD with no treatment adaptation based on interim PET results should not be considered any longer as the standard of care. In that study, PET-2 was positive in 11% of patients in the BV-AVD arm and in 14% of patients in the ABVD arm and was not used for treatment modification [29].

Two newly published studies may bring about a dramatic change in the practice of the treatment of advanced-stage HL. The randomized phase 3 HD21 trial, conducted by the GHSG, demonstrated in 1482 patients the superiority of BrECADD over EB in terms of the safety profile and non-inferiority in terms of the estimated 3-year PFS, amounting to 94.9 (95% CI 93.5–96.7) and 92.3 (95% CI 90.3–94.3), respectively [27,28]. The disease-specific death rate was 2 versus 1, with the treatment-related mortality of 0 versus 3 patients in the BrECADD versus EB arm. There were fewer episodes of neutropenia, decreased usage of blood product support, and no cases of treatment-related death. Yet, one third of the patients had positive PET-2 (Deauville score 4–5) and, therefore, received a total of 6 cycles of therapy. The 3-year PFS is the best reported to date, reaching 98% in PET-2-negative patients and 92% in PET-2-positive patients. There was no difference in OS between the two arms (98.5% in both). The other of these two studies is the randomized controlled S1826 trial by SWOG, comparing 6 cycles of nivolumab + AVD (Nivo-AVD) with BV-AVD for advanced-stage HL [26]. The study, also incorporating pediatric patients, enrolled 994 individuals at a median age of 27 years (range 12–83); 976 of them were found eligible and included in the final analysis. A quarter of the patients were younger than 18 years, and 10% were older than 60 years. A high IPS (4–7) was recorded in 32% of patients. The reported PFS (primary endpoint) at 12 months was 94% for the Nivo-AVD arm and 86% for the BV-AVD arm. Eleven deaths, including seven due to adverse events, occurred in the BV-AVD arm compared to 4 deaths in the Nivo-AVD arm, with 3 being related to adverse events. Notably, the rate of grade ≥3 hematological adverse events was higher following Nivo-AVD treatment. Likewise, a significantly higher incidence of thyroid dysfunction, with 7% hypothyroidism and 3% hyperthyroidism, was reported in the Nivo-AVD arm compared to 1% in the BV-AVD arm. At the same time, the frequency of peripheral neuropathy was 28%/4% (sensory/motor) in the Nivo-AVD arm relative to 54.2%/6.8% in the BV-AVD arm. Less than 1% of patients in that study received radiation therapy. Based on the promising findings of the study, the Nivo-AVD regimen could become a new standard treatment applicable to both pediatric and adult settings. However, a longer follow-up for treatment-related toxicity is required.

## 5. Discussion and Future Directions

**Figure 1:**
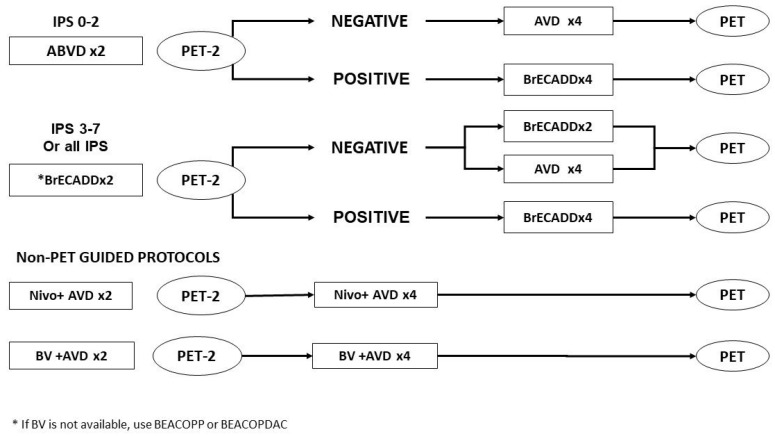
Suggested approaches to the management of advanced-stage Hodgkin lymphoma based on data from the trials incorporating novel treatment agents.

The use of ABVD as a backbone combination therapy for advanced-stage HL has been challenged in recent years. Patients with a high IPS demonstrate an inferior PFS if treated upfront with ABVD, and even dose escalation for those with a positive interim PET fails to improve the PFS rates beyond 60–67.5% (Table 1). The initiation of therapy with more aggressive regimens provides a better PFS, and an interim PET may be used as a tool for outcome prediction and decision-making regarding the reduction in the number of cycles or a change to a less toxic regimen (Figure 1).

It is hardly possible to directly compare all the discussed studies, since those initiating therapy with ABVD included patients of older age, who were excluded from the studies using the upfront EB protocol. For instance, in the RATHL study, 8% of patients were between 61 and 79 years old. Furthermore, the percentage of patients with IPS ≥ 3 was 58% in the LYSA study and 37% in the RATHL study.

In view of major advances in the management of HL patients, the issue of a long-term follow-up acquires particular significance. The recently published 5-year follow-up in the SWOG S0816 trial, analyzing pros and cons of using the PET-adapted treatment in patients with stage III/IV HL, showed that about 25% of PET-2-negative patients receiving ABVD relapsed, whereas PET-2-positive patients who were treated with EB and achieved improved PFS had increased rates of secondary malignancies (14%) [16]. Conversely, the rate of secondary primary malignancies in the PET-guided arm in the AHL2011 study, where most patients received 2 cycles of EB plus 4 cycles of ABVD, was only 2.2% at a median follow-up of 67 months [23]. These data underscore the significance of long-term follow-up in this patient population [16].

The accumulating knowledge regarding the toxicity of EB regimens has led to the efforts to minimize the number of therapeutic cycles, substitute toxic agents like procarbazine with dacarbazine (e.g., BEACOPDac) [30], and diminish the use of radiation therapy [12,13,17]. In the HD21 trial, the PFS of about 95% for the BrECADD arm is the highest reported to date, but a longer follow-up is warranted. Since the use of this protocol shows similar efficiency as EB and an improved toxicity profile due to the replacement of procarbazine with dacarbazine and omission of bleomycin, one can assume that it could safely replace EB in the treatment of patients in whom an initial aggressive protocol is beneficial. In fact, this strategy may further decrease the rate of late malignancies and attenuate the adverse ovarian effect, causing no elevation in follicle-stimulating hormone (FSH) levels and, thus, reducing premature ovarian failure. We also assume that de-escalation of therapy, as performed in the LYSA AHL2011 trial [23], is safe.

The integration of anti-PD1 checkpoint inhibitors into the first-line therapy provides outstanding initial PFS results of 94%. This regimen could be prescribed to patients older than 60 years of age; however, a longer follow-up is still required, and the substantial autoimmune toxicity, including thyroid dysfunction and peripheral neuropathy, has been reported and needs to be taken into consideration. The issue of modifying the treatment with anti-PD1 antibodies based on PET-2 results has not been fully elucidated. The fact is that the use of these drugs is known to be associated with a false positive interim PET. This may be explained by pseudo-progression or tumor flare. The delineated pitfalls in the interpretation of PET images have led to the development of the lymphoma response to immunomodulatory therapy criteria (LYRIC) by Cheson et al. [31]. Given the above, a more accurate assessment of early disease response is of major importance, and the application of additional markers such as circulating tumor DNA (ctDNA) might be beneficial [32].

Finally, to evaluate the efficiency of the emerging treatment protocols, studies focusing on the comparison of response rates and PFS in patients with high and low IPS and stage IV disease treated with these new regimens are warranted.

## 6. Conclusions

In the last decade, therapeutic strategies for the management of Hodgkin lymphoma have undergone major changes that are primarily associated with the addition of the anti-CD30 antibody drug conjugate and checkpoint inhibitors to the first-line chemotherapy. This approach has led to improved PFS and OS reported in some of the studies. Interim PET/CT is yet an efficient tool to project PFS, providing a high negative predictive value. This enables the reduction of treatment intensity through either decreasing the number of chemotherapy cycles or therapy de-escalation in cases when aggressive chemotherapy protocols were initially applied. However, false positive PET/CT results remain frequent, and this issue needs to be addressed. More accurate tools for PET/CT interpretation are warranted and could be based on the evaluation of changes in the metabolic tumor volume as well as in ctDNA and other serological markers. While the upfront use of checkpoint inhibitors is associated with promising preliminary results, longer follow-up is required. Furthermore, the significance of interim PET in predicting PFS for patients receiving regimens containing checkpoint inhibitors is not yet defined. To that end, a head-to-head comparison of BrECADD and Nivo-AVD protocols could provide the key information, contributing to the field.
